# Association of reduced heme oxygenase-1 with excessive Toll-like receptor 4 expression in peripheral blood mononuclear cells in Behçet's disease

**DOI:** 10.1186/ar2367

**Published:** 2008-01-31

**Authors:** Yohei Kirino, Mitsuhiro Takeno, Reikou Watanabe, Shuji Murakami, Masayoshi Kobayashi, Haruko Ideguchi, Atsushi Ihata, Shigeru Ohno, Atsuhisa Ueda, Nobuhisa Mizuki, Yoshiaki Ishigatsubo

**Affiliations:** 1 Department of Internal Medicine and Clinical Immunology, Yokohama City University Graduate School of Medicine, 236-0004, 3-9 Fukuura, Kanazawa-ku, Yokohama, Japan; 2 Department of Ophthalmology and Visual Science, Yokohama City University Graduate School of Medicine, 236-0004, 3-9 Fukuura, Kanazawa-ku, Yokohama, Japan

## Abstract

**Introduction:**

Toll-like receptors (TLRs) mediate signaling that triggers activation of the innate immune system, whereas heme oxygenase (HO)-1 (an inducible heme-degrading enzyme that is induced by various stresses) suppresses inflammatory responses. We investigated the interaction between TLR and HO-1 in an inflammatory disorder, namely Behçet's disease.

**Methods:**

Thirty-three patients with Behçet's disease and 30 healthy control individuals were included in the study. Expression levels of HO-1, TLR2 and TLR4 mRNA were semiquantitatively analyzed using a real-time PCR technique, and HO-1 protein level was determined by immunoblotting in peripheral blood mononuclear cells (PBMCs) and polymorphonuclear leukocytes. In some experiments, cells were stimulated with lipopolysaccharide or heat shock protein-60; these proteins are known to be ligands for TLR2 and 4.

**Results:**

Levels of expression of HO-1 mRNA were significantly reduced in PBMCs from patients with active Behçet's disease, whereas those of TLR4, but not TLR2, were increased in PBMCs, regardless of disease activity. Moreover, HO-1 expression in PBMCs from patients with Behçet's disease was repressed in the presence of either lipopolysaccharide or heat shock protein-60.

**Conclusion:**

The results suggest that upregulated TLR4 is associated with HO-1 reduction in PBMCs from patients with Behçet's disease, leading to augmented inflammatory responses.

## Introduction

Behçet's disease (BD) is an inflammatory disorder of unknown cause, characterized by recurrent oral aphthous ulcers, genital ulcers, uveitis, and skin lesions [1]. A close association of the human leukocyte antigen (HLA)-B51 allele with the disease suggests that genetic predisposition contributes to susceptibility to BD [2]. In addition, infections with agents such as herpes simplex virus [3,4] and *Streptococcus sanguis *[5] has been implicated in the development of BD, although no specific infectious agent has been identified as its cause [6]. Rather, several reports have suggested that ubiquitous antigens presented by micro-organisms, such as heat shock proteins (HSPs), trigger crossreactive autoimmune responses through molecular mimicry machinery, which results in BD [6].

Not just acquired but also innate immune systems are activated in BD, because hyperfunction of neutrophils is a hallmark of the disease [7]. However, the immunopathological mechanisms remain uncertain. Toll-like receptors (TLRs), which are expressed on phagocytes and other cells, recognize 'pathogen-associated molecular patterns' in microbes and mediate inflammatory signal transduction [8,9]. TLR2 and TLR4 recognize lipoproteins and lipopolysaccharide (LPS), respectively. Furthermore, both receptors also bind to the endogenous 60 kDa HSP (HSP60), leading to cell activation [10,11]. It is becoming clear that TLRs are involved in systemic autoimmune disorders, because it was recently demonstrated TLR2 and TLR4 are involved in rheumatoid arthritis (RA) [12-14] and TLR9 in systemic lupus erythematosus [15,16]. These findings have led to the hypothesis that microbial antigens not only trigger autoimmune responses through specific T-cell receptors but they also activate the innate immune system through the TLRs, leading to the inflammation that is characteristic of BD [17].

Few studies have been conducted to investigate the role played by the regulatory systems in inflammatory diseases of humans, including BD. We are interested in heme oxygenase (HO)-1, because accumulating evidence suggests that HO-1 protects the host in a variety of pathologic conditions [18,19]. Our laboratory has demonstrated the beneficial role of HO-1 in inflammatory lung disease [20] and lupus nephritis [21]. On the other hand, a deficiency in HO-1 expression is associated with severe chronic inflammation, as demonstrated by studies conducted in HO-1 knockout mice [22] and observations in a patient with HO-1 deficiency [23]. These findings are consistent with the notion that HO-1 plays a physiologic role in protecting against inflammation. Furthermore, our recent studies [24-26] have demonstrated substantial pathologic roles of HO-1 in rheumatic diseases. Abundant expression of HO-1 was identified in synovial tissues of patients with RA, in the absence of elevated serum HO-1 levels [24,25]. Further analysis using RA synovial cell lines suggests that HO-1 plays a regulatory role in RA inflammation [25]. Our recent study [26] showed that tumor necrosis factor (TNF) suppresses HO-1 expression in human monocytes, leading to augmentation of inflammatory responses, and that clinical efficacy of anti-TNF therapy is associated with restoration of HO-1 expression in circulating monocytes from patients with RA [26]. In another study [20], HO-1 gene therapy successfully ameliorated lung injury induced by LPS, which stimulates the innate immune system through TLR4. It is thus of interest to study the relationship between TLRs, as activating factors, and HO-1, as a regulatory factor of inflammatory responses in inflammatory disorders.

In the present study, mRNA expression levels of HO-1, TLR2, and TLR4 in circulating leukocytes from BD patients were determined. The data suggest that activation signals through essentially over-expressed TLR4 cause reduction in HO-1 expression in peripheral blood mononuclear cells (PBMC), resulting in an augmentation of inflammatory responses in BD.

## Materials and methods

### Patients and healthy donors

Thirty-three patients with BD, who met the International Study Group criteria for diagnosis of BD [27], were enrolled in the study. Their mean age was 47.7 ± 15.0 years, and 13 were male and 20 were female.

All of the patients were under the care of the Yokohama City University Hospital. As previously described [28], 13 patients with one or more lesions (including genital ulcers, uveitis, erythema nodosum, arthritis, gastrointestinal lesions, central nervous system lesions, and/or C-reactive protein >10 mg/l) were regarded to have active disease during the study.

The patients had been treated with a combination of the following agents: colchicines (17 patients), corticosteroids (13 patients), nonsteroidal anti-inflammatory drugs (14 patients), sulfasalazine (two patients), and cytotoxic drugs such as methotrexate (one patient), cyclosporine (four patients), tacrolimus (one patient) and cyclophosphamide (one patient). Thirty healthy age- and sex-matched individuals were also included as a control group. HLA-B type was determined by SRL Inc. (Tokyo, Japan) using lymphocyte cytotoxicity assay or a PCR reverse sequence specific oligonucleotides method. All experiments were conducted after written informed consent has been obtained, which was approved by the local institutional review board.

### Reagents

Reagents were obtained from the following manufactures: recombinant human TNF-α (R&D; Minneapolis, MN, USA), polymyxin B and LPS *Escherichia coli *O111: B4 (Calbiochem; La Jolla, CA, USA), low endotoxin recombinant human HSP60 (Stressgen; Victoria, Canada), and IgG_1_κ (Serotech; Oxford, UK). Infliximab was kindly provided by Tanabe Seiyaku (Osaka, Japan).

### Cell preparation and culture

PBMCs and polymorphonuclear leukocytes (PMNs) were isolated by centrifugation over two Ficoll-Hypaques gradients of specific gravities 1.077 (ICN; Aurora, OH, USA) and 1.119 (Nacalai; Kyoto, Japan). Purity of the separated neutrophils, which were determined by flow cytomeric scattergram, was typically above 97% [7]. Monocytes were negatively selected by magnetic cell sorting (Miltenyi Biotec; Gladbach, Germany) using a monocyte isolation kit (Miltenyi Biotec). More than 95% of the obtained monocytes expressed CD14, based on flowcytomeric analysis [26].

The cells were incubated in hepes modified RPMI1640 (Sigma-Aldrich; Saint Louis, MO, USA) containing 10% fetal calf serum (Equitech-bio; Kerrville, TX, USA), 2 mmol/l L-glutamine (Sigma-Aldrich), 100 U/ml penicillin plus 100 μg/ml streptomycin (Sigma-Aldrich) in a 5% carbon dioxide in an air incubator at 37°C. To determine HO-1 expression at mRNA and protein levels, cells were cultured in the presence or absence of LPS (10 ng/ml) or HSP60 (3 μg/ml) for 6 to 24 hours.

### Transfection

Purified monocytes (1 × 10^6^) were transfected with 3 μg of human HO-1 expression vector or control vector by using Nucleofector (Amaxa Biosystems; Gaithersburg, MD, USA) and human monocyte Nucleofector kit (Amaxa Biosystems) [25,26]. Twenty four hours later, the cells were used for further experiments.

### Reverse transcription PCR and Real-time PCR

Total RNA was isolated from cells with TRIzol reagent (Invitrogen, Carlsbad, CA, USA) [21,24-26]. One microgram of total RNA served as a template for single-stranded cDNA synthesis in a reaction using oligo (dT) primers and SuperScript II (Invitrogen). For the reverse transcription PCR, 1 μl cDNA was incubated with 9.375 μl de-ionized distilled water, 2 μl dNTP, 2.5 μl 10 × PCR buffer, 0.125 μl Taq polymerase (Takara, Ohtsu, Japan), and primer pairs for target genes. The primers used in the study are summarized in Table 1.

**Table 1:** Primers used in the study

Primer	Sense/antisense	Sequence
HO-1	Sense	CAGGCAGAGAATGCTGAG
	Antisense	GCTTCACATAGCGCTGCA
TLR2	Sense	TGACTGCTCGGAGTTCTCCC
	Antisense	GTCAGCACCAGAGCCTGGAG
TLR4	Sense	GCGGCTCGAGGAAGAGAAGA
	Antisense	AGGCTCTGATATGCCCCATC
GAPDH	Sense	ACAGTCAGCCGCATC
	Antisense	AGGTGCGGCTCCCTA
TNF-α	Sense	ATGAGCACTGAAAGCATGATC
	Antisense	GGCGATGCGGCTGATGGT
CD14	Sense	CGGCCGAAGAGTTCACAAGT
	Antisense	AGTGCAGTCCTGTGGCTTC
MD-2	Sense	TAAATCTTTTCTGCTTACTGA
	Antisense	TACTCAATTTATTCTAATTTGAAT

HO, heme oxygenase; MD, Myeloid differentiation factor-2 ; TLR, Toll-like receptor; TNF, tumor necrosis factor; GAPDH, glyceraldehyde-3-phosphate dehydrogenase.

Cycling conditions included 35 cycles of amplification for 30 seconds at 94°C, 30 seconds at 55°C, 1 minute at 72°C, and a final extension phase consisting of one cycle of 10 minutes at 72°C. The primers and probes for human HO-1, TLR2, TLR4, CD14, TNF-α, MD-2 (Myeloid differentiation factor-2), and glyceraldehyde-3-phosphate dehydrogenase (GAPDH) used in the real-time PCR were purchased from PE Applied Biosystems (Foster City, CA, USA). Real-time PCR was performed using a BD Qtaq DNA polymerase (BD Bioscience), and the data were analyzed by the ABI prism 7700 sequence detection system (PE Applied Biosystems, Franklin Lakes, NJ, USA). Briefly, 1/50 of cDNA derived from 1 μg total RNA, 200 nmol/l probe, and 800 nmol/l primers were incubated in 25 μl at 50°C for 2 minutes and 95°C for 10 minutes, followed by 40 cycles of 95°C for 15 seconds and 60°C for 1 minute. The analysis system determined the number of cycles at which the amplified DNA in the sample exceeded the threshold during the PCR. Gene expression levels of the individual samples were calculated on standard curves of each cDNA generated by serial dilutions of the PCR amplified products. The data on HO-1, TLR2, TLR4, and TNF-α were standardized to the expression of GAPDH in the same samples, using multiplex PCR technique. Expression level of HO-1 mRNA in a sample is indicated as arbitrary units.

### Immunoblot analysis

The expression of HO-1 protein was determined by immunoblotting as described previously [25]. Briefly, cells were treated with lysis buffer (137 mmol/l NaCl, 20 mmol/l Tris-HCl, 50 mmol/l NaF, 1 mmol/l EDTA, and Triton-X), supplemented with a protease inhibitor cocktail (Sigma-Aldrich) for 30 minutes on ice, and the supernatants were recovered by centrifugation at 15,000 rpm for 30 minutes. For TLR2 and TLR4 immunoblotting, after addition of lysis buffer, cells were homogenized for 15 minutes by ultrasonifier (Branson Japan, Kanagawa, Japan). The samples were resolved electrophoretically on a 4% to 20% gradient of polyacrylamide gel (Daiichi Kagaku, Tokyo, Japan) and transferred onto a polyvinyldene difluoride membrane (Millipore, Billerica, MA, USA). After blocking with 5% skimmed milk/Tris-buffered saline overnight at 4°C, the membrane was incubated with optimally diluted anti-HO-1 monoclonal antibody (Stressgen), anti-TLR2 and anti-TLR4 (Imgenex, San Diego, CA, USA) monoclonal antibody, or anti-actin goat polyclonal antibody (Santa Cruz Biotechnology, Santa Cruz, CA, USA) for 1 hour at room temperature or overnight at 4°C, and subsequently for 45 minutes with horseradish peroxidase-conjugated anti-mouse secondary antibody (Amersham Life Sciences, Piscataway, NJ, USA) or rabbit anti-goat IgG horseradish peroxidase conjugate (Zymed, South San Francisco, CA, USA). The signals were developed by using the enhanced chemiluminescence detection system (Amersham Life Sciences). The amount of blotted protein was measured densitometrically by using Scion image analysis and image processing software (NIH Image Engineering, Bethesda, MD, USA).

### Statistical analysis

Mann-Whitney U-test, Kruskal-Wallis test with post-hoc Scheffe's test, paired *t*-test, and regression analysis were used to test for differences. *P *values less than 0.05 were considered statistically significant.

## Results

### Reduced HO-1 mRNA expression in PBMCs from patients with active BD

**Figure 1:**
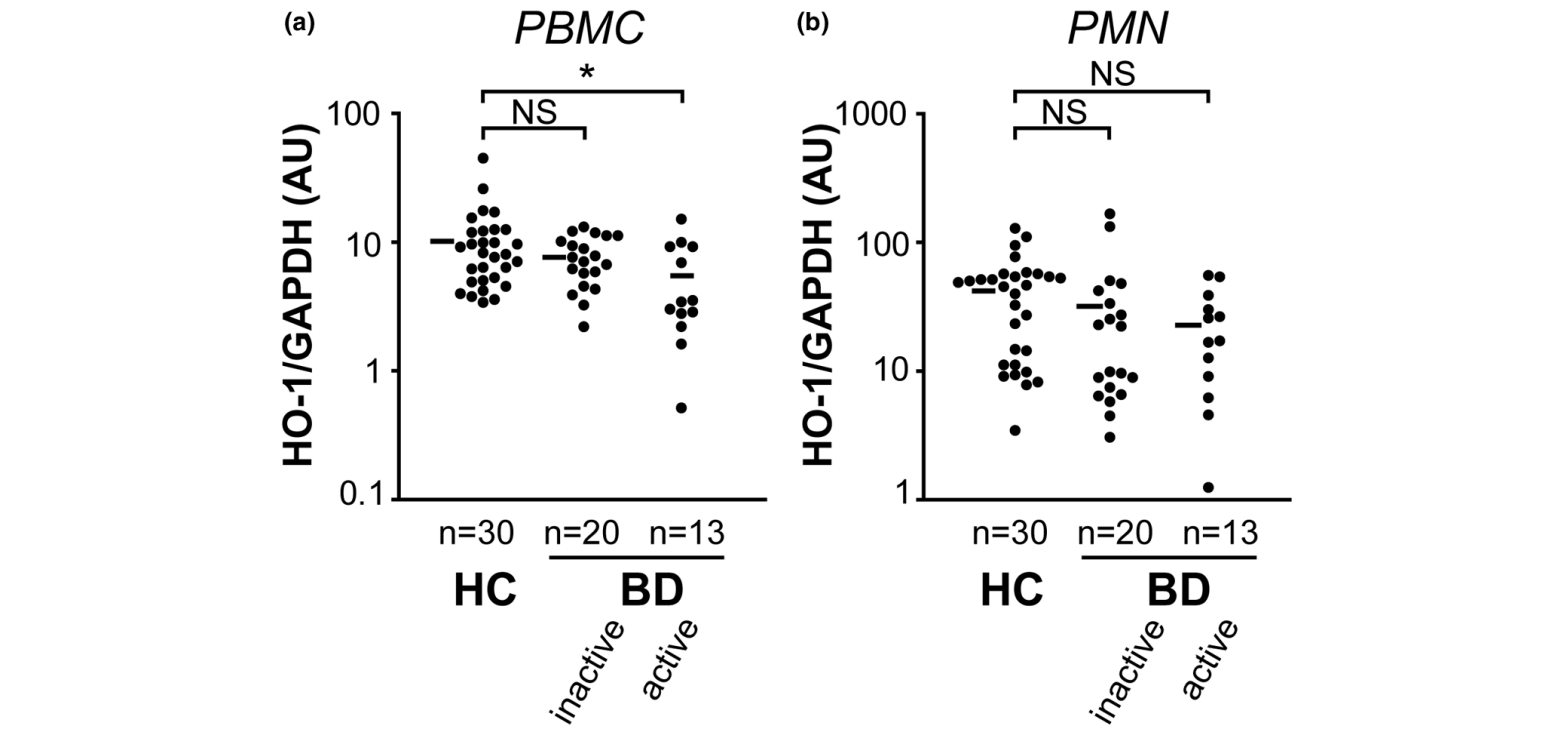
HO-1 mRNA expression in PBMCs and PMNs from patients with BD. **(a) **Peripheral blood mononuclear cell (PBMC) heme oxygenase (HO)-1 mRNA expression in healthy controls (HC), and patients with active and inactive Behçet's disease (BD) were determined semiquantitatively by real-time PCR. Horizontal bars represent mean values of HO-1 mRNA. **(b) **Polymorphonuclear leukocyte (PMN) HO-1 mRNA expression levels of HC, and patients with active and inactive BD. *P < 0.05, as determined using the Kruskal-Wallis test with post-hoc Scheffe's test. AU, arbitrary unit; GAPDH, glyceraldehyde-3-phosphate dehydrogenase; NS, not significant.

HO-1 mRNA expression level was determined in circulating leukocytes from BD patients by using a real-time PCR technique (Figure 1). A good correlation between HO-1 mRNA and protein levels has been demonstrated [26]. Consistent with previous findings [24], we found no significant difference in HO-1 mRNA expression in PBMCs between BD patients (including both patients with active and those with inactive disease) and healthy control individuals (data not shown). A more detailed analysis based on disease activity, however, revealed that PBMCs from patients with active BD, but not those with inactive disease, expressed significantly lower HO-1 mRNA levels than did PBMCs from healthy control individuals (Figure 1a). Because HO-1 is preferentially expressed by monocytes among PBMCs, amounts of HO-1 mRNA may depend on the proportion of monocytes detected [26]. CD14 mRNA levels determined by real-time PCR were comparable between BD and healthy control individuals, indicating that there was no difference between groups in the proportion of monocytes among circulating leukocytes (data not shown). Moreover, no significant difference was found in absolute counts of monocytes between patients with active BD and those with inactive disease (active 497.9 ± 218.8/μl versus inactive 462.7 ± 182.4/μl; *P *= 0.77, by Mann-Whitney U-test), indicating that HO-1 expression was reduced in individual cells from patients with active disease. As shown in Figure 1b, HO-1 mRNA levels in PMNs were not significantly different between BD patients and control individuals (Figure 1b).

No particular clinical manifestations, including ocular lesions (Table 2) and treatments (data not shown), were associated with the reduction in HO-1 mRNA expression in PBMCs. There were no differences in mRNA expression levels of HO-1 and TLRs between HLA-B51-positive and -negative patients (Table 2). Levels of HO-1 mRNA expression were not altered by treatment with colchicine or prednisolone in the patients (data not shown).

**Table 2:** HO-1 mRNA expression in patients with BD

	PBMCs/PMNs	Ocular involvement		HLA-B51	
		
		- (*n *= 17)	+ (*n *= 16)	- (*n *= 10)	+ (*n *= 14)
HO-1 (AU)	PBMCs	7.7 ± 3.1	5.7 ± 4.1	8.1 ± 3.9	5.6 ± 3.7
	PMNs	32.9 ± 46.1	31.6 ± 34.1	31.6 ± 38.0	19.9 ± 18.0

Values are expressed as mean ± standard deviation. AU, arbitrary unit; BD, Behçet's disease; HLA, human leukocyte antigen; HO, heme oxygenase; PBMC, peripheral blood mononuclear cells; PMN, peripheral blood multinuclear cells.

### Increased TLR4, but not TLR2, expression by PBMCs from BD patients

**Figure 2:**
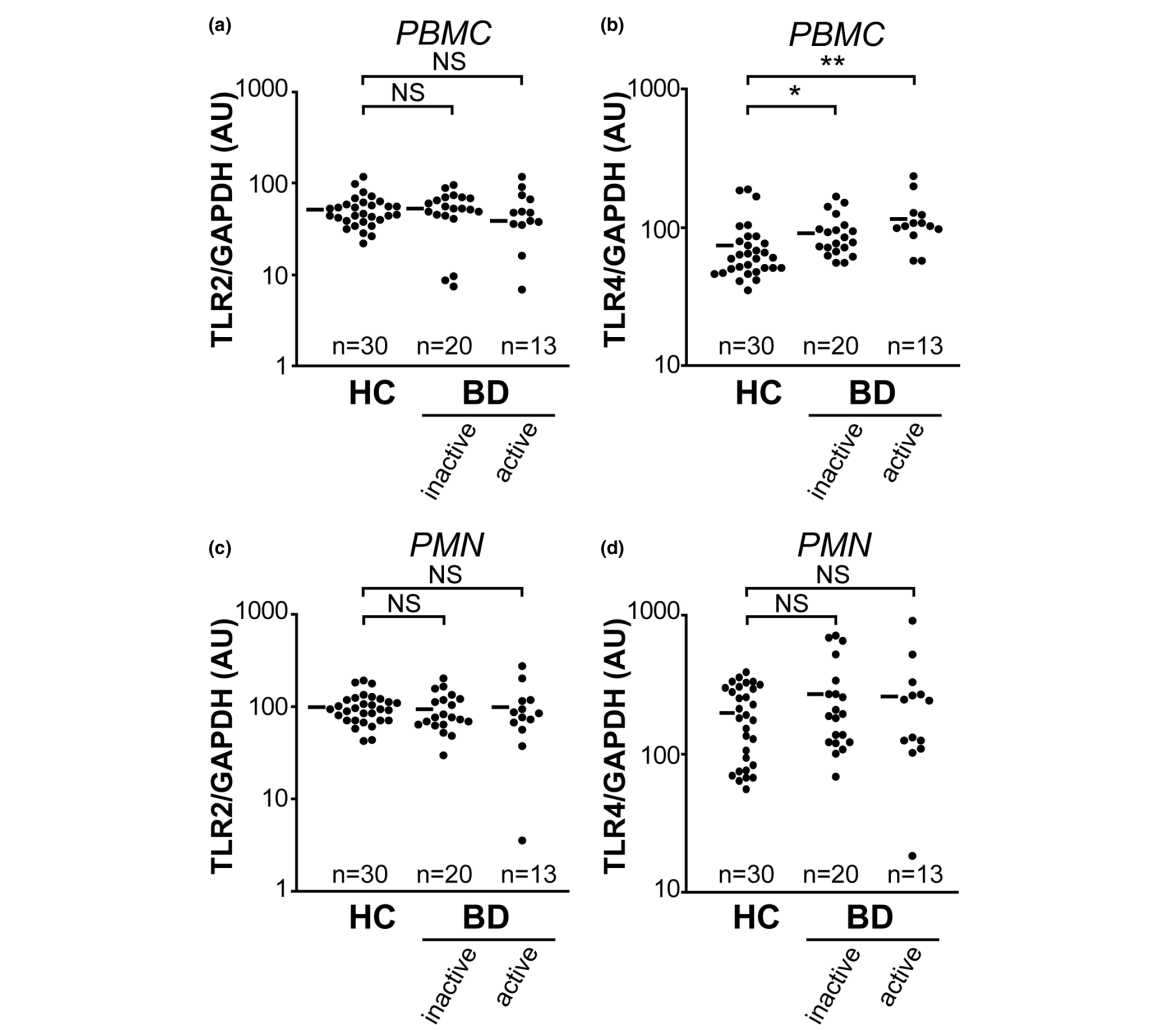
TLR2 and TLR4 mRNA expression in PBMCs and PMNs from patients with BD. Expression levels in peripheral blood mononuclear cells (PBMCs) of **(a) **Toll-like receptor (TLR)2 and **(b) **TLR4 mRNA in healthy controls (HC), and patients with active and inactive Behçet's disease (BD) were determined semiquantitatively by real-time PCR. Horizontal bars represent mean values of HO-1 mRNA. Expression levels in polymorphonuclear leukocytes (PMNs) of **(c) **TLR2 and **(d) **TLR4 in HC, and patients with active and inactive BD. *P < 0.05, **P < 0.01, as determined using the Kruskal-Wallis test with post-hoc Scheffe's test. AU, arbitrary unit; GAPDH, glyceraldehyde-3-phosphate dehydrogenase; NS, not significant.

Because HSP60 has been implicated in the pathogenesis of BD [17], levels of mRNA for TLR2 and TLR4 (both of which recognize HSP60 as a ligand) were examined in PBMCs and PMNs from patients with BD (Figure 2). In preliminary experiments, the relationship between levels of TLR mRNA and protein in circulating leukocytes was examined. Briefly, after fractionating PBMCs into CD14-positive cells and CD14-depleted cells by means of magnetic cell sorting, mRNA and protein levels of TLRs were compared by using real-time PCR and immunoblotting techniques, respectively. TLR4 was preferentially expressed on CD14-positive cells, but not CD14-depleted cells, at both mRNA and protein levels (Additional file 1). Moreover, TLR4 and TLR mRNA levels correlated well with protein levels.

No significant differences were found in levels of TLR2 mRNA expression in PBMCs between patients with active BD, patients with inactive BD, and healthy control individuals (Figure 2a). On the other hand, TLR4 mRNA expression levels were elevated in PBMCs from patients, irrespective of disease activity (Figure 2b) and HLA-B51 phenotype (data not shown). However, no significant differences in levels of mRNA expression for CD14 and MD-2, which are critically involved in LPS-mediated signal transduction of TLR, were found between patients and control individuals (data not shown). There was no abnormality in mRNA expression of TLR2 and TLR4 in PMNs (Figure 2c,d). The results indicate that TLR4 mRNA expression is constitutively increased in PBMCs from BD patients.

### Inverse correlation between HO-1 and TLR4 mRNA in PBMCs from BD patients

**Figure 3:**
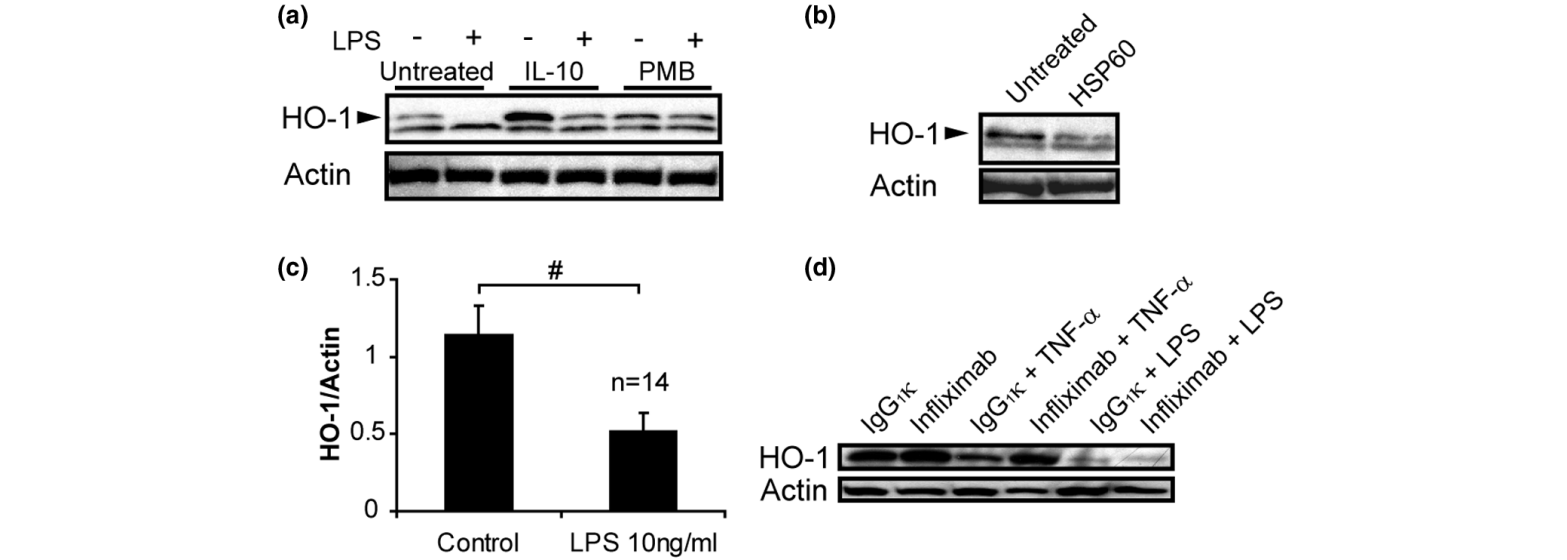
Effects of HSP60 and LPS on HO-1 protein expression in PBMCs from patients with BD. **(a) **Effect of lipopolysaccharide (LPS) stimulation on heme oxygenase (HO)-1 and actin protein expression in peripheral blood mononuclear cells (PBMCs) from patients with Behçet's disease (BD). PBMCs from a BD patient were stimulated with LPS in the presence or absence of 10 ng/ml interleukin (IL)-10 and 100 μg/ml polymyxin B (PMB). Representative immunoblotting data for HO-1 protein in the cells are shown. The arrowhead indicates 32 kDa molecular weight HO-1 specific band. **(b) **Effect of heat shock protein (HSP)60 (3 μg/ml) stimulation on endogenous HO-1 protein expression in PBMCs from patients with BD. The arrowhead indicates 32 kDa HO-1 specific band. A representative of three independent experiments is shown. **(c) **Mean and standard error of the mean (SEM) values of HO-1 and actin protein expression in PBMCs stimulated by LPS (1 ng/ml) for 24 hours in patients with BD (*n *= 14). ^#^*P *< 0.001, as determined using paired *t*-test. **(d) **Effect of infliximab (10 μg/ml) or IgG_1_κ (10 μg/ml) on HO-1 expression in LPS (10 ng/ml) or tumor necrosis factor (TNF; 1 ng/ml) treated PBMCs.

**Figure 4:**
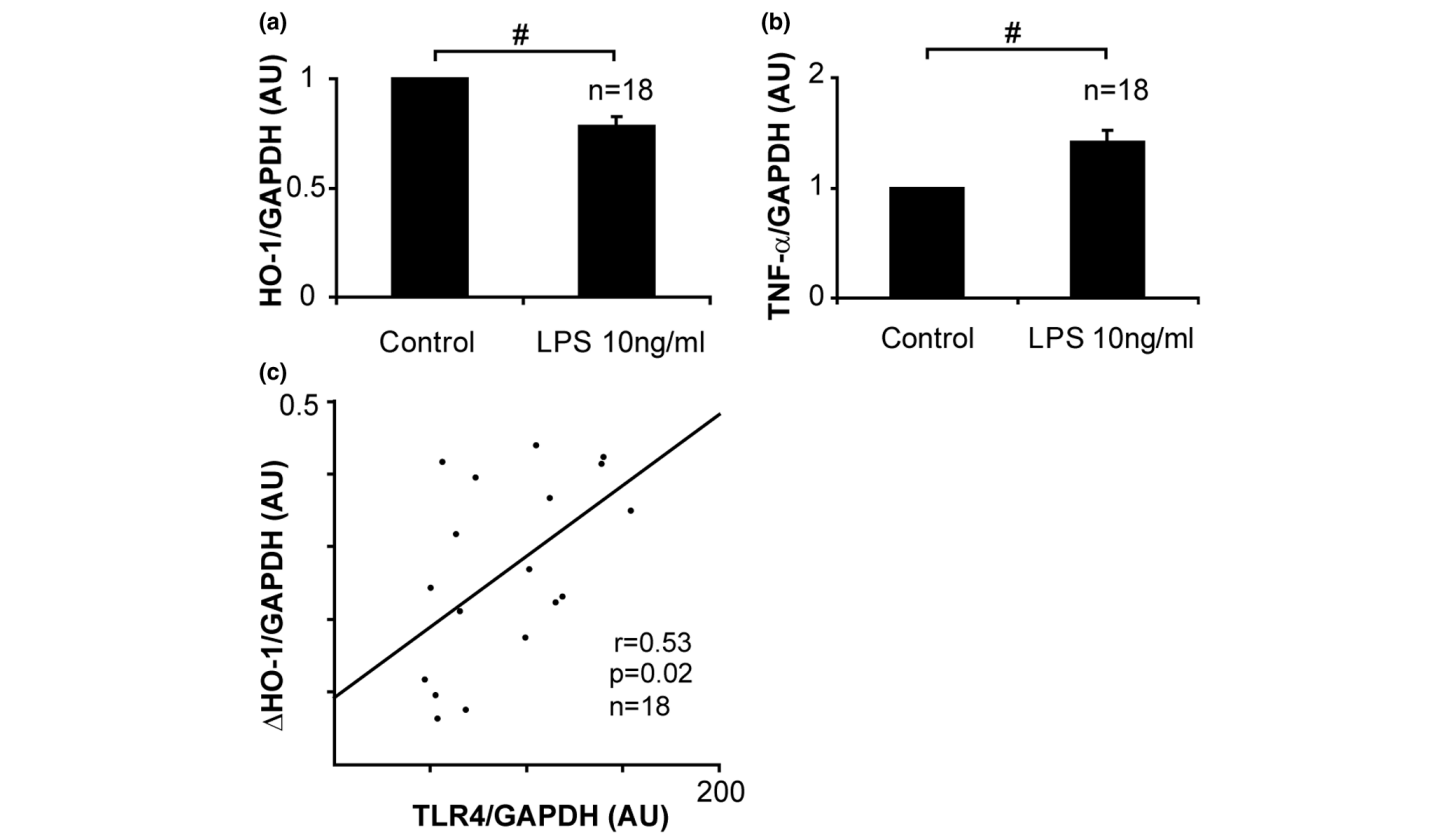
Effect of LPS on HO-1 mRNA expression in PBMCs from BD patients. Expression of **(a) **heme oxygenase (HO)-1 and **(b) **tumor necrosis factor (TNF) mRNA in peripheral blood mononuclear cells (PBMCs) from patients with Behçet's disease (BD; *n *= 18). Values presented are mean and standard error of the mean (SEM) change, regarding 1 to be the value of untreated cells. ^#^*P *< 0.001, as determined using paired *t*-test. **(c) **Relationship of endogenous Toll-like receptor (TLR)4 mRNA with gap in HO-1 mRNA between PBMCs subjected to 6 hours of treatment with lipopolysaccharide (LPS) and untreated PBMCs (ΔHO-1). *P *= 0.02, *r *= 0.53, as determined by regression analysis. AU, arbitrary unit; GAPDH, glyceraldehyde-3-phosphate dehydrogenase.

TLR4 signaling triggers activation of the innate immune system, whereas HO-1 plays a regulatory role in inflammatory response. Analysis of the relationship between the two molecules showed that TLR4 mRNA was inversely correlated with HO-1 mRNA in PBMCs from BD patients (Additional file 2; *P *< 0.05, *r *= -0.42, by regression analysis). Because LPS (a TLR4 ligand) has been shown to suppress interleukin-10-dependent HO-1 expression in human PBMCs [29], it is plausible that excessively expressed TLR4 contributes to defective HO-1 expression in PBMCs from BD patients. As expected, the immunoblotting study revealed that stimulation with LPS reduced HO-1 expression in PBMCs from patients with BD, irrespective of the presence or absence of interleukin-10 (Figure 3a). The suppressive effect on HO-1 expression was completely abrogated by a LPS neutrizer, namely polymyxin B (Figure 3a). Similarly, real-time PCR analysis revealed a reduction in HO-1 mRNA levels in LPS-stimulated PBMCs (Figure 4a) when TNF mRNA expression was elevated (Figure 4b). The magnitude of LPS-induced HO-1 suppression (calculated as the gap in HO-1 mRNA between PBMCs subjected to 6 hours of LPS treatment and untreated PBMCs [ΔHO-1]) was significantly correlated with TLR4 mRNA expression levels in untreated PBMCs (Figure 4c).

In our previous study [26] we demonstrated that TNF enhances HO-1 mRNA degradation, resulting in a reduction in HO-1 expression in human monocytes. Because TLR4 signaling leads to synthesis of TNF, which may be involved in the reduction in HO-1 expression in PBMCs from patients with BD. However, levels of TNF mRNA did not correlate with those of HO-1 in PBMCs from patients with BD (data not shown). Moreover, although the preliminary experiments confirmed that 10 ng/ml LPS efficiently stimulated PBMCs to produce substantial amounts of TNF protein, anti-TNF-α antibody infliximab did not eliminate the suppressive effect of LPS on HO-1 expression in monocytes *in vitro *(Additional file 3). The findings indicated that the effect is not solely dependent on TNF (Figure 3d).

### No effect of forced HO-1 expression on TLR2 and TLR4 mRNA in human PBMCs

**Figure 5:**
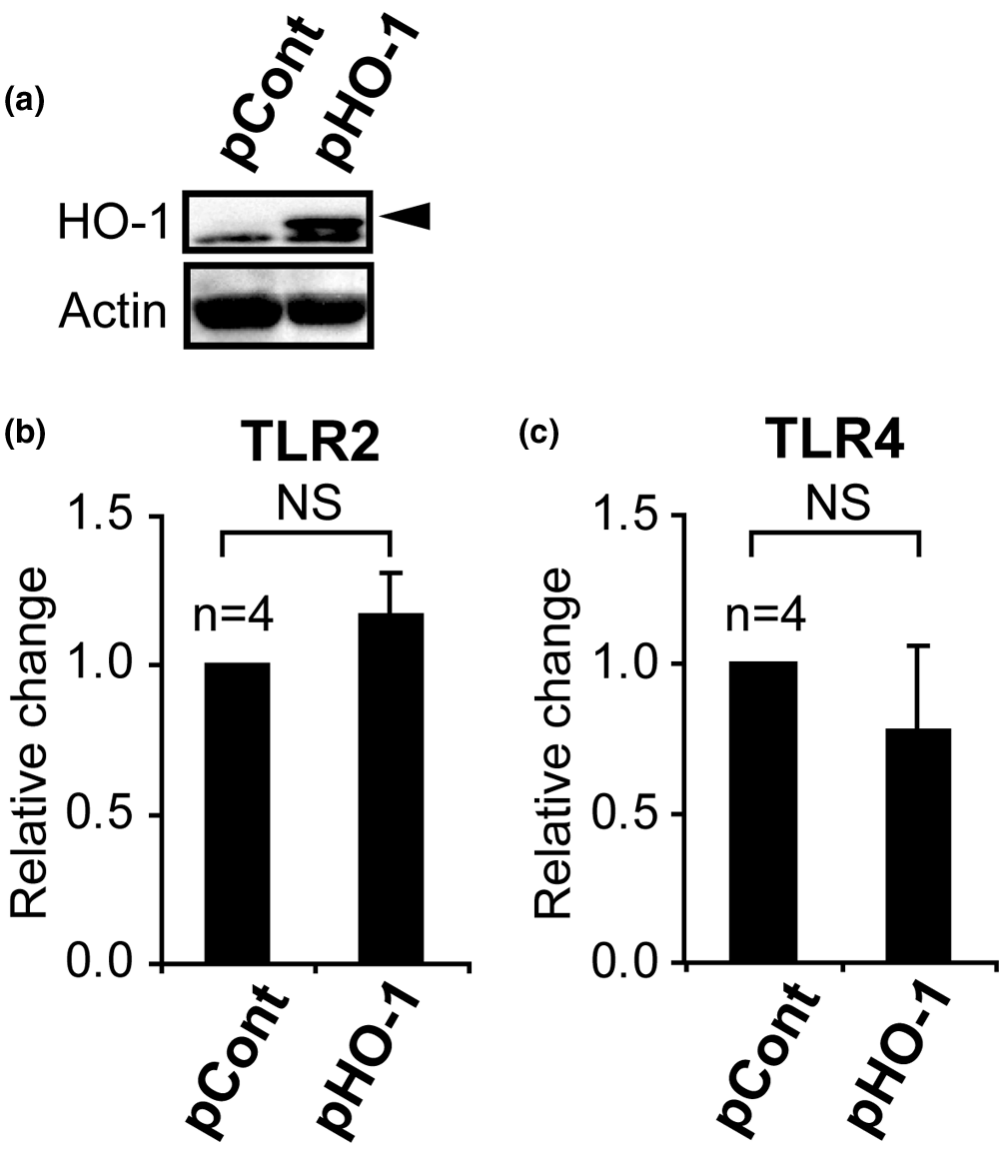
Effect of forced HO-1 expression on Toll-like receptor (TLR)2 and TLR4 mRNA expression in peripheral monocytes. **(a) **Immunoblotting analysis of heme oxygenase (HO)-1 and actin in pHO-1 (human HO-1 expression vector) or pCont (control vector) transfected monocytes. The arrow represents HO-1 protein. **(b) **Real-time PCR analysis of TLR2 and TLR4 mRNA expression in pHO-1 transfected peripheral blood mononuclear cells (PBMCs). NS, not significant.

Because our previous study demonstrated bidirectory interactions between HO-1 and TNF [26], we also examined effects of HO-1 upregulation on TLR levels in monocytes. Over-expression of HO-1 protein was confirmed by immunoblotting analysis 24 hours after transfection with pHO-1 (human HO-1 expression vector) into monocytes. Under these conditions, no differences were found in expression levels of TLR2 and TLR4 between HO-1 cDNA transfected monocytes and controls (Figure 5). Taken together, our findings implicate the involvement of excessive TLR4 expression in low levels of HO-1 mRNA expression in PBMCs from patients with BD.

## Discussion

In the present study we found endogenous HO-1 expression to be decreased in PBMCs from patients with active BD. Dysregulation of HO-1 expression is associated with some rheumatic diseases. Our previous studies [24,25] have demonstrated elevated serum HO-1 levels in patients with adult onset Still's disease and hemophagocytic syndrome, and aberrant expression of HO-1 in synoviocytes from patients with RA. However, reduced HO-1 levels in leukocytes have not been demonstrated in other rheumatic diseases. Evidence suggests that increased expression of HO-1 can benefit the host in a variety of pathologic conditions, including inflammatory changes, whereas a deficiency in HO-1 expression is associated with vigorous inflammation, as demonstrated by studies of HO-1 knockout mice and observed in a patient with HO-1 deficiency [22,23]. In RA, HO-1-expressing cells were located in the lining and sublining layers, but not in the cartilage-pannus junction, where bone and cartilage are actively destroyed [25,30,31]. Furthermore, our previous report [26] demonstrated that selective knockdown of HO-1 expression by using specific small interfering RNA resulted in upregulation of synthesis of proinflammatory cytokines, including interleukin-6, interleukin-8 and TNF, which have been shown to be elevated in sera from BD patients [6]. This suggests that leukocyte function is regulated by HO-1 expressed in the cells [26]. Thus, defective expression of HO-1 may be involved in the inflammation characteristic of BD, especially in patients with active disease.

Although a pathogenic role of anti-HSP60 specific autoimmune responses has been suggested in BD, abnormal activation of the innate immune system has also been identified in the disease [1,6]. Furthermore, involvement of TLRs has been shown in other systemic autoimmune diseases [16]. In the present study, expression levels of TLR2 and TLR4 were examined because both TLRs recognize HSP60 as ligands [10,11]. Actually, HSP60 was reported to be expressed in PBMCs, and in intestinal and mucocutaneous lesions from BD patients [32,33]. Our findings demonstrated that levels of TLR4 mRNA, but not of TLR2 mRNA, are constitutively increased in PBMCs from patients with BD, regardless of disease activity. The data suggest possible involvement of TLR4 in BD, although TLR4 has been also implicated in other rheumatic diseases [13,34]. Abnormal expression of TLR4 can predispose to defective HO-1 expression in BD PBMCs, because TLR4 may be a putative HO-1 repressor in hepatic ischemia/reperfusion injury mouse model [35]. Indeed, HO-1 expression was suppressed in PBMCs stimulated with LPS [29]. Moreover, elevated soluble CD14 in plasma of BD patients may further facilitate LPS binding to TLR4 [36]. Interestingly, LPS-induced lung injury in a mouse model was rescued by administration of an HO-1 adenovirus vector [20]; this suggests that HO-1 supplementation may have utility as a strategy for countering TLR4-related inflammation. Such a strategy may also be applicable to BD.

TNF plays a critical role in the development of BD [1,37,38]. Several studies, including ours, have demonstrated that TNF is excessively produced in patients with active BD [28,38]. Indeed, anti-TNF therapy is effective in the disease, especially for management of ocular lesions [39]. In our previous study [26] we showed that TNF suppresses HO-1 expression levels in human peripheral monocytes, thereby accelerating inflammatory responses; this suggests that excessive TNF levels contribute to defective HO-1 expression. However, no association was found between HO-1 and TNF mRNA levels in circulating PBMCs from patients with BD. In addition, the suppressive effect of LPS on HO-1 was not abrogated by anti-TNF antibody, at least *in vitro*, although significant synthesis of TNF in response to LPS was confirmed in the experiments (Additional file 3). These data, rather, suggest that the effect of LPS is mainly mediated by a pathway distinct from TNF. However, TNF may also contribute to defective HO-1 expression *in vivo*, because other types of cells also produce TNF in BD. Taken together, our findings suggest that highly expressed TLR4 might contribute to reduced HO-1 expression, leading to an activation of the innate immune system in BD, although other factors including TNF may be involved in the defective HO-1. Because TLRs other than TLR4 are also likely to be involved in the pathogenesis BD [17], further investigation of molecular mechanisms, including interactions between TLRs and HO-1, are required, especially those that distinguish BD from other inflammatory diseases.

## Conclusion

Based on the data presented, we hypothesize that HSP60 stimulates not only antigen-specific autoimmune responses but also the innate immune system through constitutively over-expressed TLR4, which mediates HO-1 reduction in PBMCs, leading to inflammation in BD. Restoration of HO-1 expression might be a promising therapeutic strategy in the disease. Alternatively, specific intervention in TLR4-mediated signals that lead to HO-1 reduction may also be of benefit in BD.

## Abbreviations

BD = Behçet's disease; GAPDH = glyceraldehyde-3-phosphate dehydrogenase; HLA = human leukocyte antigen; HO = heme oxygenase; HSP = heat shock protein; LPS = lipopolysaccharide; PBMC = peripheral blood mononuclear cell; PCR = polymerase chain reaction; PMN = polymorphonuclear leukocyte; RA = rheumatoid arthritis; TLR = Toll-like receptor; TNF = tumor necrosis factor.

## Competing interests

The authors have received no financial support or other benefits from commercial sources for the work reported here, and the authors have no other financial interests that could create a potential conflict of interest or the appearance of a conflict of interest with regard to the present study.

## Authors' contributions

YI designed and organized the study. YK, MT, RW, SM, and MK conducted the laboratory work. YK, MT, RW, SM, MK, AI, HI, SO, AU, NM, and YI were involved in the analysis and interpretation of data. YK, MT, and YI were involved in writing the report. All authors read and approved the final manuscript. The authors thank Mr Tom Kiper for his review.

## Supplementary Material

Additional file 1 The Protein and mRNA TLR2, TLR4 and HO-1 expression levels in PBMCs. (A) TLR2, TLR4, HO-1, and actin protein expression in PBMCs and CD14^+/- ^cells from a healthy control individual (HC). (b) TLR2, TLR4, CD14, and β-actin mRNA expression levels in CD14^+/- ^cells from HCs. (C) Correlation between densitometrically analyzed HO-1 protein levels and semiquantatively evaluated HO-1 mRNA expression by real-time PCR in PBMCs and CD14^+/- ^cells from a HC. Click here for file

Additional file 2 The correlation between HO-1 and TLR4 mRNA levels in PBMCs from patients with BD. Click here for file

Additional file 3 The effect of LPS, PMB, and infliximab on TNF-α production by PBMCs. TNF-α levels in supernatants of PBMC-cultured media recovered after 24 hours of stimulation with LPS, with or without PMB and/or infliximab, as determined by ELISA. Click here for file
