# Supplementary figures and images for: Association of reduced heme oxygenase-1 with excessive Toll-like receptor 4 expression in peripheral blood mononuclear cells in Behçet's disease
Source: Arthritis Res Ther. 2008 Jan 31;10(1):R16. doi: 10.1186/ar2367 (PMC2374472; doi:10.1186/ar2367)

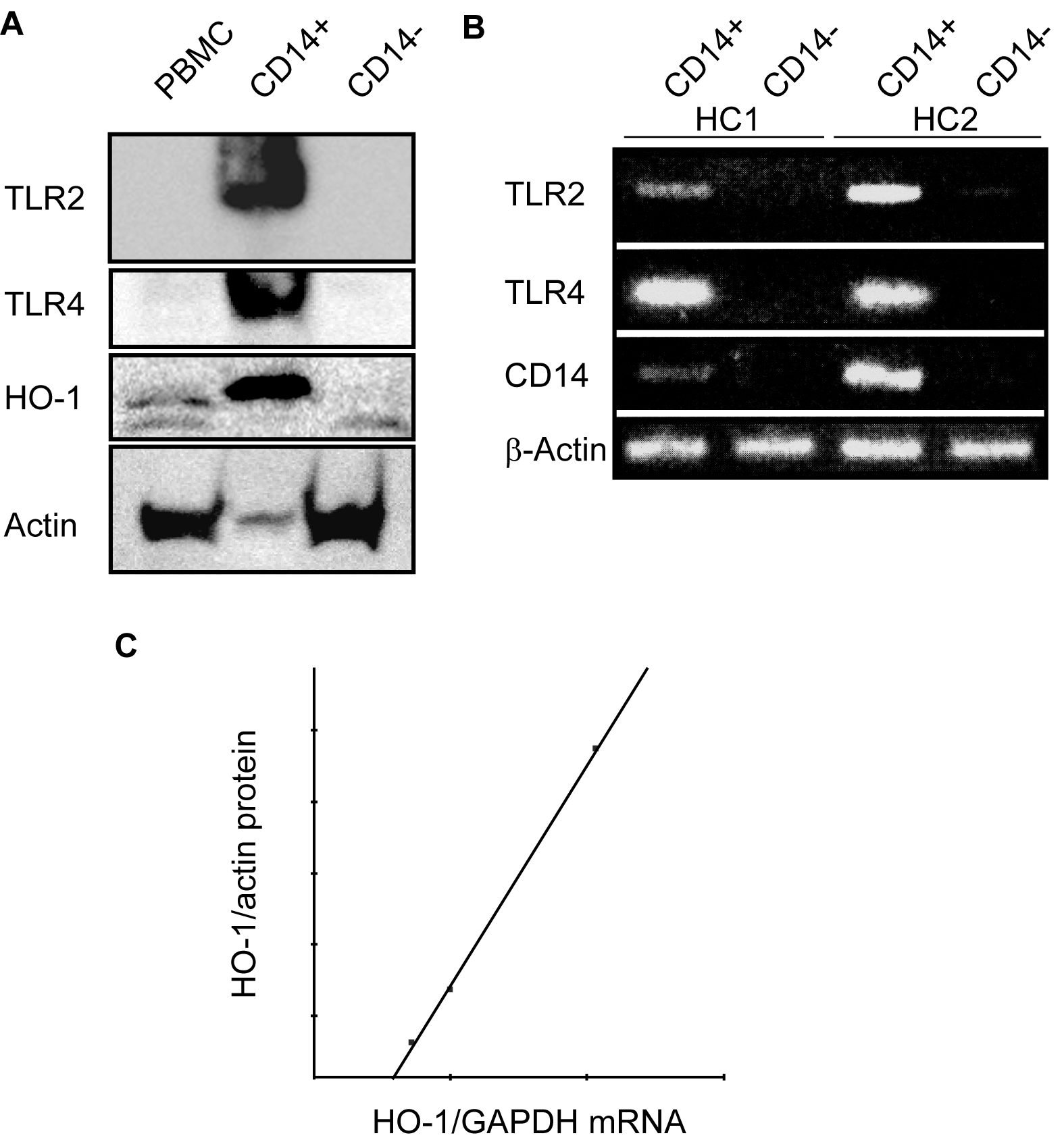

Supplement: Additional file 1 — The Protein and mRNA TLR2, TLR4 and HO-1 expression levels in PBMCs. (A) TLR2, TLR4, HO-1, and actin protein expression in PBMCs and CD14+/- cells from a healthy control individual (HC). (b) TLR2, TLR4, CD14, and β-actin mRNA expression levels in CD14+/- cells from HCs. (C) Correlation between densitometrically analyzed HO-1 protein levels and semiquantatively evaluated HO-1 mRNA expression by real-time PCR in PBMCs and CD14+/- cells from a HC. [file ar2367-S1.TIFF]

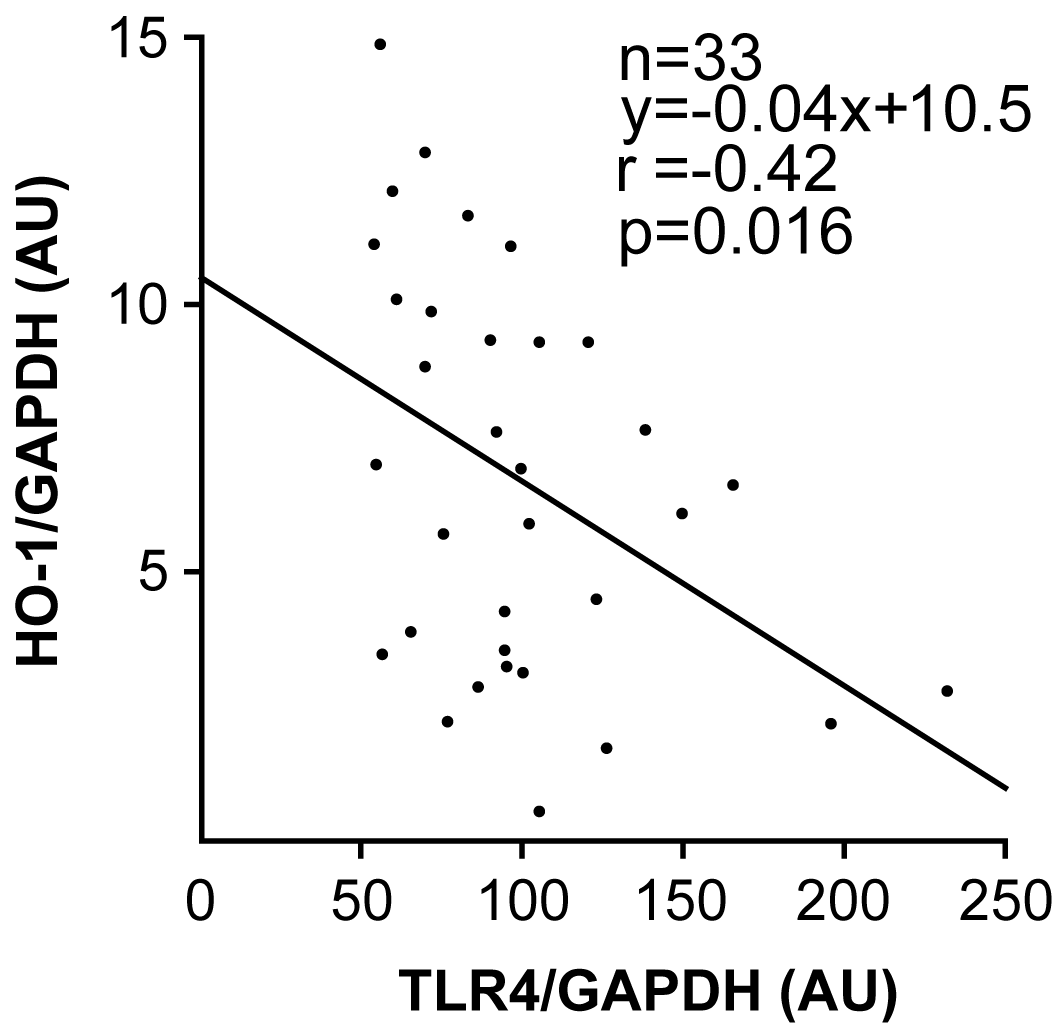

Supplement: Additional file 2 — The correlation between HO-1 and TLR4 mRNA levels in PBMCs from patients with BD. [file ar2367-S3.TIFF]

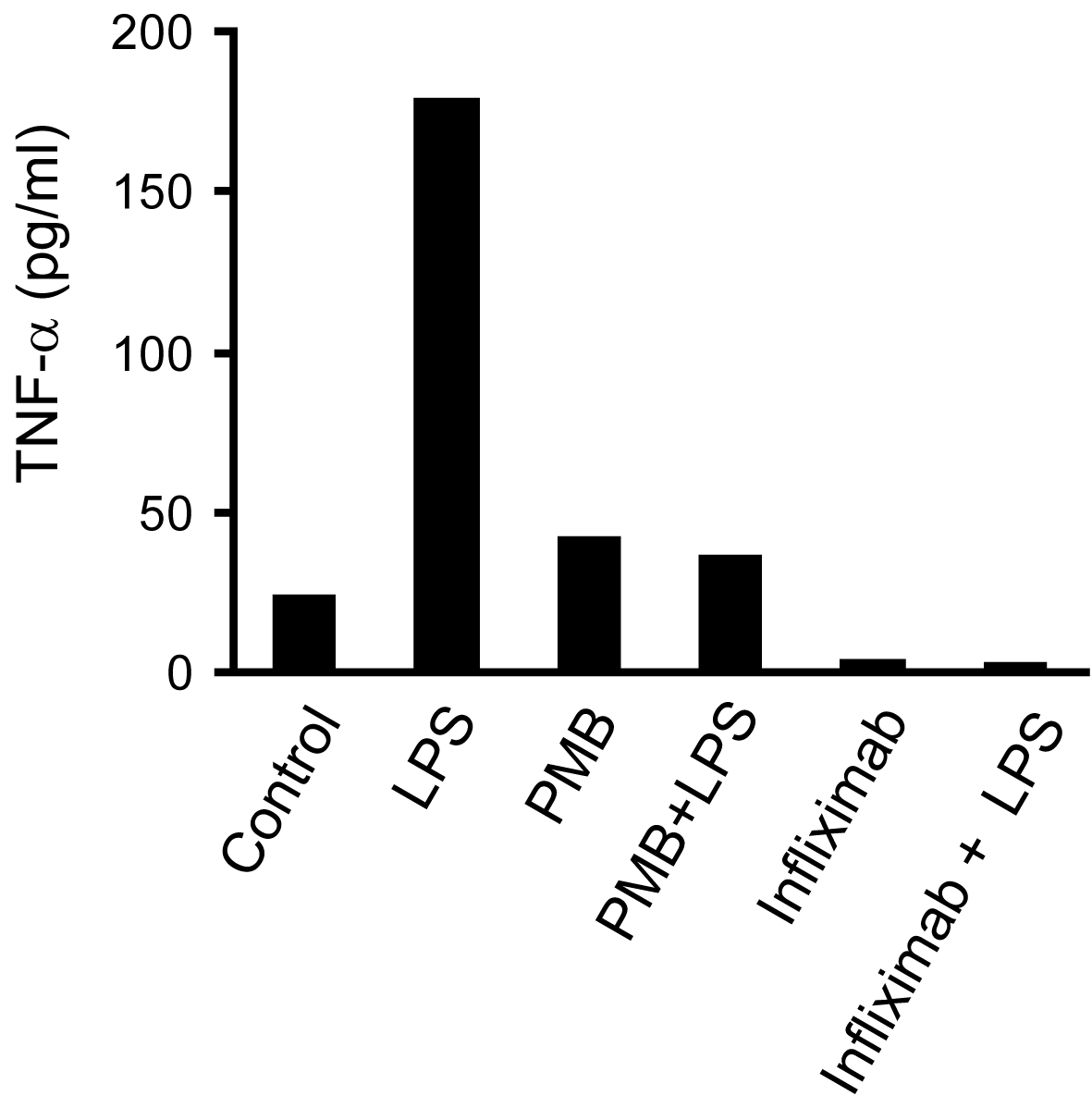

Supplement: Additional file 3 — The effect of LPS, PMB, and infliximab on TNF-α production by PBMCs. TNF-α levels in supernatants of PBMC-cultured media recovered after 24 hours of stimulation with LPS, with or without PMB and/or infliximab, as determined by ELISA. [file ar2367-S2.TIFF]
